# Treatment recommendations for recurrent vulvovaginal candidiasis across complementary and conventional disciplines: a national survey of Australian healthcare professionals

**DOI:** 10.1007/s11096-026-02165-5

**Published:** 2026-05-26

**Authors:** Rommy Everingham-Gordon, Elizabeth Steels, Cathy J. Watson, Kathryn J. Steadman

**Affiliations:** 1 School of Pharmacy and Pharmaceutical Sciences, Faculty of Health, Medicine and Behavioural Sciences, The University of Queensland, Brisbane, QLD Australia; 2 Centre for Epidemiology and Biostatistics, Melbourne School of Population and Global Health, The University of Melbourne, Carlton, VIC Australia; 3 Women’s Health Clinics, The Royal Women’s Hospital, Parkville, VIC Australia

**Keywords:** Candidiasis, Complementary medicines, Clinical decision-making, Herbal medicine, Probiotics, Vulvovaginal

## Abstract

**Introduction:**

Management of recurrent vulvovaginal candidiasis remains challenging. It is treated by healthcare professionals across disciplines including general practice, pharmacy, and complementary medicine. Conventional antifungal regimens supported by formal treatment guidelines are the standard of care. No standardised guidance exists for complementary medicine, and the treatments used in contemporary practice remain largely undocumented.

**Aim:**

To identify treatments recommended for recurrent vulvovaginal candidiasis across healthcare disciplines and the sources used to inform clinical decisions.

**Method:**

A national cross-sectional, open, anonymous, self-administered online survey of Australian healthcare professionals from conventional and complementary medicine disciplines was conducted between November 2022 and September 2023 using a 19-item questionnaire. The questionnaire captured treatment recommendations for recurrent or persistent *Candida*-related vulvovaginal symptoms, including conventional and complementary medicines, over-the-counter products, and lifestyle strategies such as dietary and hygiene advice, and information sources used in diagnosis and management.

**Results:**

A total of 223 healthcare professionals from 13 disciplines met the inclusion criteria, comprising 68 conventional medicine and 155 complementary medicine practitioners. Overall, 122 different treatments were identified, including 69 distinct herbal medicines, reflecting substantial variation in clinical recommendations across disciplines. Conventional medicines were recommended by 43.9% of respondents, herbal medicines by 65.0%, and nutrient-based interventions by 86.1%. Oral fluconazole and intravaginal clotrimazole were the most frequently recommended conventional agents. Probiotics, administered either orally or intravaginally, were cited across professional groups. Frequently recommended herbal medicines included *Allium sativum* (garlic), *Handroanthus impetiginosus* (pau d’arco), and *Pseudowintera colorata* (horopito). Common lifestyle strategies included reducing dietary sugar, alcohol moderation, avoiding synthetic underwear, and modifying post-toileting hygiene practices. Information sources also differed by discipline, with conventional medicine practitioners more often consulting the Therapeutic Guidelines (eTG) and complementary medicine practitioners more commonly consulting peer-reviewed journal articles.

**Conclusion:**

Practitioner-reported recurrent vulvovaginal candidiasis management included a broad range of treatment strategies across healthcare disciplines. The heterogeneity of recommendations, together with overlap across disciplines, supports further evaluation of selected treatments and evidence-informed cross-disciplinary guidance to support safer, more coordinated care.

**Supplementary Information:**

The online version contains supplementary material available at https://doi.org/10.1007/s11096-026-02165-5.

## Impact statements

- These findings document the treatment approaches recommended in day-to-day practice for recurrent vulvovaginal candidiasis, including conventional antifungals, probiotics, nutritional and herbal medicines, and non-pharmacological advice.
- The variability in information sources used by practitioners highlights a need for evidence-informed resources that support shared clinical decision-making across professional groups. Strengthening guidance on therapies such as probiotics and selected complementary medicines suggests opportunities to improve decision support tools for primary care practitioners recommending complementary and integrative therapies.
- Awareness of treatment patterns supports safer, more coordinated interdisciplinary care and assists healthcare providers to address concurrent product use, monitor potential interactions, and facilitate open, informed treatment planning with patients.

## Introduction

Recurrent vulvovaginal candidiasis (RVVC) is a condition that continues to pose therapeutic challenges in clinical practice. Affecting over 100 million women globally each year, with peak prevalence among those aged 25 to 34 years [1], RVVC arises from *Candida* species overgrowth attributed to disturbances in vaginal microbial balance [2, 3]. Although antibiotic exposure, chronic inflammation, impaired immune function, and more recently host genetic factors have been linked to recurrent disease susceptibility in some populations [4–7], RVVC remains aetiologically unpredictable, with some cases lacking identifiable triggers [8, 9]. The global clinical significance of *Candida* species has been formally recognised, with the World Health Organization (WHO) classifying them among its fungal priority pathogens and issuing updated guidance relevant to the clinical management of vaginal infections and related conditions [10, 11].

While antifungal pharmaceuticals remain the standard of care, recurrence and drug resistance are common [4, 12]. Care for RVVC has been characterised by diagnostic uncertainty, variable practitioner knowledge, and suboptimal treatment outcomes [13–16], factors that may contribute to women seeking support from multiple healthcare providers, including complementary medicine practitioners [15, 17, 18]. Complementary medicine refers to a broad array of medicinal and healthcare disciplines that fall outside the scope of conventional medicines, including traditional systems of medicine, herbal therapies, nutritional supplements, and functional foods [19, 20]. In this study, complementary medicines referred to herbal medicines and nutritional medicines including vitamins, minerals, amino acids and functional foods, used for therapeutic intent in clinical care.

Complementary medicines are used in the management of RVVC, as either primary treatments or adjunctive strategies [17, 21–23]. However, the specific treatments recommended in Australian clinical settings remain poorly characterised, despite ongoing use of complementary approaches by women with recurrent vaginal symptoms [17, 23] and broader international recognition of complementary and integrative medicine in healthcare [23, 24]. While recent work has reviewed different clinical considerations for RVVC [25], there remains limited documentation of which treatments, both pharmaceutical and complementary, are selected and applied in everyday clinical practice.

### Aim

This study aimed to identify treatments recommended for RVVC across healthcare disciplines, and to examine the sources used to inform these recommendations. The goal was to provide a clear, practice-based overview of practitioner-led treatment patterns and interprofessional perspectives to inform future evidence-based guidance and support safe, well-informed coordinated treatment planning.

## Method

This article has been prepared in accordance with the Checklist for Reporting Results of Internet E-Surveys (CHERRIES) [26, 27].

### Study design and participants

A cross-sectional, descriptive study was conducted between November 2022 and September 2023 as an open, anonymous, self-administered online survey of healthcare professionals practising in Australia. Using a 19-item questionnaire, the study captured practitioner treatment strategies including recommendations for complementary and conventional medicines.

Electronic consent was obtained from all participants using a digital Participant Information and Consent Form. The study collected no identifying information, and participants were free to exit at any time. No incentives were offered. The 19-item questionnaire was delivered using Checkbox software, and addressed three major content areas: practitioner demographics, treatment recommendations, and information sources consulted for diagnosis and management. Treatment items captured recommendations used for recurrent or persistent *Candida*-related vulvovaginal symptoms. All questions required a response; however, a *prefer not to answer* option was available where applicable.

Treatment-related items covered conventional, herbal and nutritional therapies; non-prescription over-the-counter (OTC) products and home remedies; and adjunctive lifestyle-based strategies including dietary modification and hygiene advice. The term *recommend,* rather than *prescribe,* was used to reflect the varying professional scopes of practice and capture the full range of therapeutic guidance offered in clinical care. Predefined response options were derived from: (a) treatments listed in guidelines for conventional medicines [11, 28, 29]; (b) herbal medicines and nutrients described in the literature [30–45]; (c) non-prescription products commonly found in Australian pharmacies or complementary practice (for OTC and home-remedies); and (d) dietary and lifestyle strategies cited in the literature in the context of RVVC management [46–48]. Free text boxes enabled additional responses. Respondents were also asked if they had personally experienced RVVC. A standalone descriptive question was included to explore whether respondents generally recommend complementary medicines alongside conventional medicines in the management of RVVC. This item was designed to elicit a broader view of clinical strategy and decision-making, beyond individually selected treatments. Responses were recorded using a three-category scale: most of the time (≥ 50%), sometimes (< 50%), or never (0%).

Two rounds of pilot testing were undertaken. Five health professionals sourced through professional networks reviewed questionnaire functionality. A further seven practitioners (naturopaths, pharmacists, and traditional Chinese Medicine (TCM) clinicians) sourced through practitioner networks provided feedback to refine language and flow. Estimated completion time was approximately five minutes. Pilot responses were not retained in the final dataset. No statistical validation was undertaken, consistent with the exploratory purpose of the questionnaire.

Eligible participants were healthcare professionals from conventional and complementary medicine disciplines, practising in Australia, who provided clinical advice, recommendations, or prescriptions for the management of RVVC. Using snowballing and convenience sampling, the questionnaire was distributed via professional associations, practitioner networks, retail health outlets, and conferences. Online promotion was conducted through social platforms such as Instagram, Facebook, and LinkedIn to achieve broad national reach across regions and disciplines. As an exploratory study, a formal power calculation was not required; recruitment continued until survey closure to maximise reach and diversity across disciplines.

### Data analysis

Survey data were exported to Microsoft Excel for cleaning and verification, then analysed descriptively using IBM SPSS Statistics. All submissions meeting eligibility were included, including partial completions, provided they contained a professional background and at least one treatment recommendation. No correction was applied for missing data. Participation and completion rates were calculated based on survey link access, survey initiation, and the proportion of respondents who completed the final questionnaire item [26, 27]. IP matching was used to identify potential duplicates, which were then cross-checked against demographic characteristics (e.g. age, professional background, years in practice, gender); only the most complete record was retained.

Demographic characteristics and treatment responses were reported descriptively, with categorical variables expressed as counts and percentages, and age reported both categorically and as a mean (SD). Percentages for each questionnaire item were calculated using denominators consistently restricted to respondents who provided a substantive answer relevant to the research question. Responses such as *prefer not to answer* or *I do not recommend* were excluded to ensure denominators reflected only actionable recommendations. All responses were manually cross-checked for coding accuracy and recorded as binary indicators (1 = selected; 0 = not selected). Text responses were incorporated into relevant response item counts where applicable and required no interpretive coding.

### Ethics approval

Ethics approval was granted on 22 August 2022 by The University of Queensland Human Research Ethics Committee (2022/HE001188).

## Results

Of the 405 survey page visits, 251 proceeded to enter data, yielding a participation rate of 62.0%. After exclusion of submissions without a stated profession (n = 9), no treatment recommendations (n = 16), or duplicate submissions (n = 3), 223 responses met inclusion criteria. Of these eligible respondents, 207 completed the questionnaire in full (92.8%).

Participant characteristics are summarised in Table 1. Thirteen professions were represented, classified as either conventional medicine (n = 68; 30.5%) or complementary medicine (n = 155; 69.5%) practitioners. Complementary medicine practitioners formed the majority of the sample. Two participants selected “Other” (a sports therapist and a researcher) but described substantial clinical experience and submitted comprehensive complementary medicine treatment recommendations for RVVC; both were accordingly included in the complementary medicine category.

**Table 1:** Demographic characteristics of health professionals who met the study inclusion criteria (n = 223). Percentages are based on the number of respondents who answered the questionnaire item

Categorisation	n	%
Gender (n = 205)		
Women	184	89.8
Men	21	10.2
Non-binary	0	0.0
Age (years) (n = 186)		
20–34	45	24.2
35–44	50	26.9
45–54	46	24.7
55 +	45	24.2
Conventional medicine (n = 68)		
Medical doctor/General medical practitioner (GP)	24	35.3
Integrative medical practitioner	2	2.9
Gynaecologist	8	11.8
Pharmacist	25	36.8
Nurse/nurse practitioner	6	8.8
Sexual health physician	3	4.4
Complementary medicine (n = 155)		
Naturopath	118	76.1
Clinical nutritionist	16	10.3
Herbal medicine practitioner	6	3.9
Traditional Chinese medicine practitioner	12	7.7
Homeopath	1	0.6
Sports therapist	1	0.6
Researcher	1	0.6
Years in Practice (n = 210)		
0–5 years	51	24.3
6–10 years	39	18.6
11–15 years	35	16.7
16–20 years	15	7.1
21 + years	70	33.3
Practice Setting (n = 204)		
Sole practitioner (exclusively)	69	33.8
Group practice (with other healthcare professionals)	79	38.7
Online/telehealth (exclusively)	1	0.5
Multiple streams	55	27.0
Practice/Work location (n = 210)		
Metro (exclusively)	132	62.9
Rural (exclusively)	52	24.8
Remote (exclusively)	8	3.8
Multiple region (across more than one region)	18	8.6
Australian State or Territory (n = 208)		
Australian Capital Territory	5	2.4
New South Wales	41	19.7
Northern Territory	4	1.9
Queensland	47	22.6
South Australia	13	6.3
Tasmania	6	2.9
Victoria	71	34.1
Western Australia	21	10.1

The majority of participants (89.8%; 184/205) identified as female. The overall mean age of respondents reporting age was 45.2 years (SD = 12.4). Clinical experience varied, with 24.3% (51/210) reporting five years or fewer in practice, and one-third (33.3%; 70/210) reporting 21 years or more. Most respondents (62.9%; 132/210) practised in metropolitan areas, while 8.6% (18/210) engaged across multiple regions, including metropolitan–rural and rural–remote (Table 1).

Across the full dataset, 122 different treatments were identified and classified into four groups: 10 conventional medicines, 69 herbal medicines (comprising single-herb agents and multi-herb formulations, either traditional or commercially prepared), 21 nutritional medicines or functional food-based treatments, and 22 additional treatments, such as non-prescription items and home remedies. Figures presented in the tables reflect only those respondents who made recommendations within each category; full treatment listings are provided in Online Resource 1.

Conventional medicines were recommended by 43.9% of respondents (98/223). Of these 68.4% (67/98) were conventional medicine practitioners, and 31.6% (31/98) were complementary medicine practitioners. Oral fluconazole was the most cited medicine (67.3%; 66/98), followed closely by intravaginal clotrimazole (63.3%; 62/98), with fewer respondents listing intravaginal boric acid and topical clotrimazole (Table 2). Among conventional medicine practitioners, oral fluconazole was most frequently recommended (86.6%), while among complementary medicine practitioners, intravaginal boric acid was most frequently recommended (48.4%).

**Table 2:** Conventional medicines recommended by study participants for the management of RVVC

Conventional medicines	Respondents recommending conventional medicine n = 98		Conventional medicine practitioners n = 67		Complementary medicine practitioners n = 31	
	n	%	n	%	n	%
Fluconazole oral capsule	66	67.3	58	86.6	8	25.8
Clotrimazole pessary or cream (intravaginal)	62	63.3	49	73.1	13	41.9
Boric acid capsule (intravaginal delivery)	32	32.7	17	25.4	15	48.4
Clotrimazole topical external cream (external/vulval application)	31	31.6	25	37.3	6	19.4
Nystatin cream (intravaginal)	23	23.5	17	25.4	6	19.4
Miconazole cream (intravaginal)	9	9.2	8	11.9	1	3.2
Itraconazole oral capsule	5	5.1	4	6.0	1	3.2
Miconazole cream (external/vulval application)	3	3.1	3	4.5	0	0.0
Amphotericin lozenge (intravaginal delivery)	2	2.0	2	3.0	0	0.0
Nystatin oral	1	1.0	1	1.5	0	0.0

Herbal medicines were recommended by 65.0% of respondents (145/223). A total of 69 herbal medicines were reported: 10 drawn from predefined response options and 59 contributed as original entries by respondents. Herbal medicines recommended by at least 10 participants are displayed in Table 3. The most frequently reported herbs were garlic (55.2%; 80/145), pau d’arco (48.3%; 70/145), and horopito (47.6%; 69/145).

**Table 3:** Herbal medicines recommended for the management of RVVC by more than 10 study participants

Herbal medicines	Respondents recommending herbal medicines n = 145		Conventional medicine practitioners n = 4		Complementary medicine practitioners n = 141	
	n	%	n	%	n	%
Garlic (*Allium sativum*)	80	55.2	1	25.0	79	56.0
Pau d'arco (*Handroanthus impetiginosus*)	70	48.3	0	0.0	70	49.6
Horopito (*Pseudowintera colorata*)	69	47.6	1	25.0	68	48.2
Cranberry (*Vaccinium macrocarpon*)	31	21.4	3	75.0	28	19.9
Curcumin (*Curcuma longa*)	22	15.2	0	0.0	22	15.6
St Mary's Thistle (*Silybum marianum*)	22	15.2	0	0.0	22	15.6
Green tea (*Camellia sinensis*)	16	11.0	0	0.0	16	11.3
Oregano (*Origanum vulgare*)	16	11.0	0	0.0	16	11.3
Calendula (*Calendula officinalis*)	13	9.0	0	0.0	13	9.2
Chamomile (*Matricaria chamomilla*)	13	9.0	0	0.0	13	9.2
Ginger (*Zingiber officinale*)	13	9.0	0	0.0	13	9.2
Tea tree (*Melaleuca alternifolia*)	13	9.0	0	0.0	13	9.2
Rosemary (*Rosmarinus officinalis*)	11	7.6	0	0.0	11	7.8
Pomegranate (*Punica granatum*)	11	7.6	0	0.0	11	7.8

Nutrient-based interventions were recommended by 86.1% of respondents (192/223) (Table 4). Oral intake of probiotics was the most cited treatment, selected by 94.8% (182/192), followed by zinc (48.4%), vitamin D (34.4%), and vitamin C (32.3%).

**Table 4:** Vitamins, minerals, amino acids, and functional foods recommended by more than 10 study participants for the management of RVVC

Nutritional medicine/functional food	Respondents recommending nutritional medicines n = 192		Conventional medicine practitioners n = 41		Complementary medicine practitioners n = 151	
	n	%	n	%	n	%
Probiotics (oral)	182	94.8	36	87.8	146	96.7
Zinc	93	48.4	2	4.9	91	60.3
Vitamin D	66	34.4	7	17.1	59	39.1
Vitamin C	62	32.3	3	7.3	59	39.1
Yoghurt (oral)	53	27.6	10	24.4	43	28.5
N-acetylcysteine	45	23.4	1	2.4	44	29.1
Lactulose (oral)	13	6.8	1	2.4	12	7.9

Of the OTC products and home remedies reported, the most frequently recommended was the intravaginal use of probiotic products (71.9%; 120/167). pH-balancing gels, yoghurt, and vinegar preparations, applied either intravaginally or topically, were each recommended by 15–20% of respondents (Table 5).

**Table 5:** Over-the-counter products and home remedies recommended by more than 10 participants for the management of RVVC

OTC Products/home remedies	Respondents recommending OTC/home remedies n = 167		Conventional medicine practitioners n = 34		Complementary medicine practitioners n = 133	
	n	%	n	%	n	%
Probiotics (intravaginal application)	120	71.9	12	35.3	108	81.2
pH balance wash or gel (intravaginal application e.g. Aci-Jel Balance)	34	20.4	18	52.9	16	12.0
Yoghurt (intravaginal application)	34	20.4	1	2.9	33	24.8
Yoghurt external/vulval	31	18.6	0	0.0	31	23.3
Vinegar intravaginal (e.g. Apple cider vinegar)	25	15.0	0	0.0	25	18.8
Vinegar external/vulval (e.g. Apple cider vinegar)	25	15.0	1	2.9	24	18.0
Intimate wash (external application e.g. Femfresh, Canestan, Vagisil, Summer’s Eve)	21	12.6	7	20.6	14	10.5
Moisture replacement (intravaginal application e.g. Aci-Jel Restore, Replens, Vagisil Pro-Hydrate)	20	12.0	9	26.5	11	8.3
Vaginal douche (intravaginal application e.g. Summer’s Eve)	16	9.6	1	2.9	15	11.3
Lactulose intravaginal	12	7.2	1	2.9	11	8.3

**Fig. 1:**
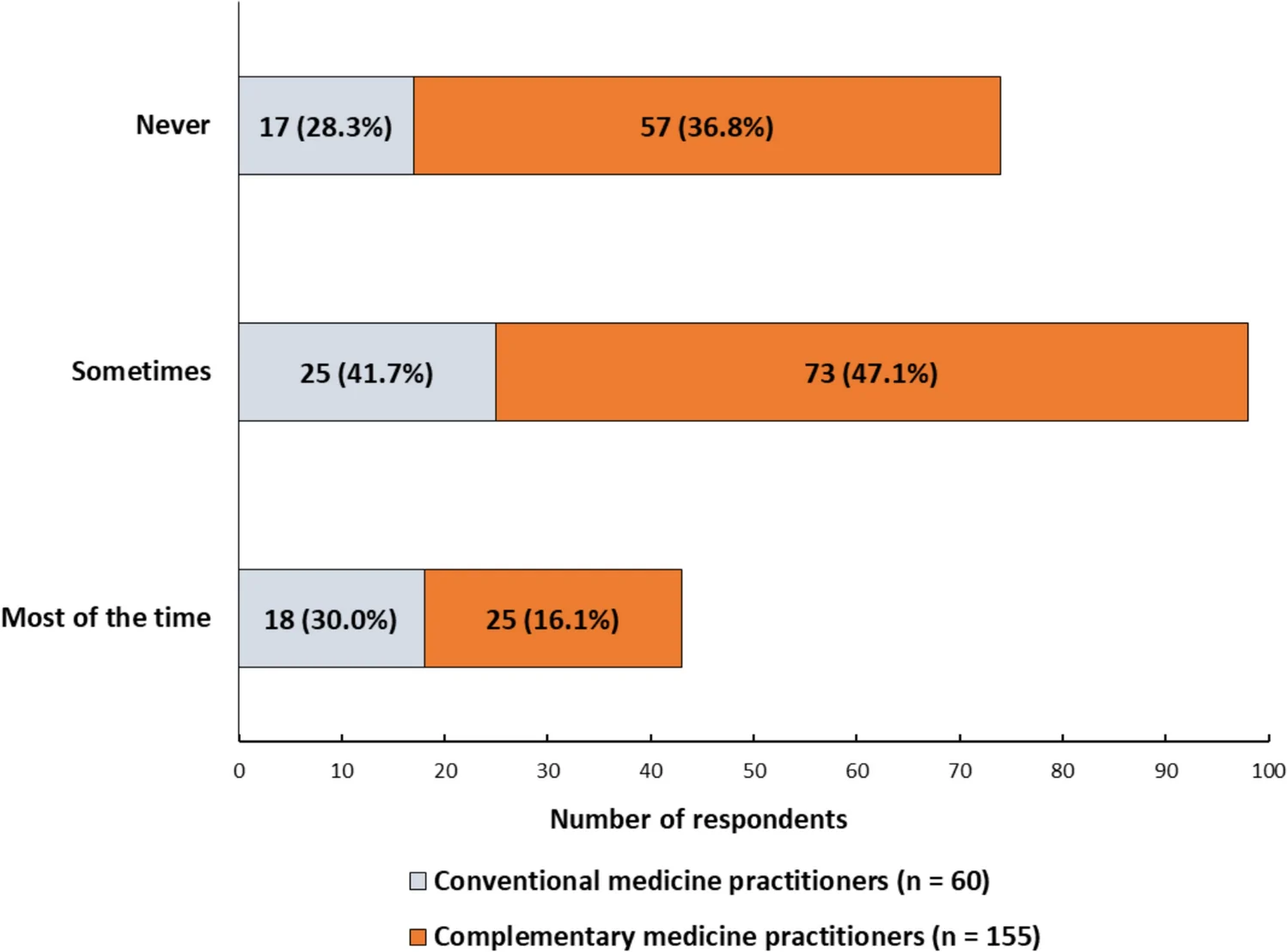
Responses to the item: “Do you recommend conventional medicines along with complementary medicines or functional foods specifically for the treatment of recurrent vulvovaginal candidiasis?” Responses were recorded using a 3-point scale. Percentages were calculated using the number of respondents within each practitioner group (complementary medicine: n = 155; conventional medicine: n = 60) to account for unequal group sizes

No differences in treatment recommendations were observed between female respondents with (n = 85) and without (n = 98) self-reported RVVC. In the overall sample, responses to the broader descriptive question on whether practitioners recommend conventional medicines along with complementary medicines indicated that 65.6% (141/215) do so either most of the time or sometimes (Fig. 1).

Dietary changes were recommended by 79.8% of respondents, and most commonly involved reducing sugar and refined carbohydrates (99.4%), moderating or eliminating alcohol (80.3%), and restricting potential allergens such as dairy or wheat (64.6%). Hygiene-related strategies included avoidance of synthetic underwear (87.9%), limiting excessive vulval washing (69.6%), and wiping backward after urination (64.3%).

Participants consulted multiple information sources to guide the diagnosis and treatment of RVVC. Conventional medicine practitioners cited the Therapeutic Guidelines (eTG) (57.6%), journal articles (42.4%), and the Australian STI Management Guidelines (32.2%). Complementary medicine practitioners more commonly referenced peer-reviewed journal articles (73.8%), along with textbooks (59.7%) and clinical reference databases such as the Natural Medicines Database (46.3%) and Integrative Medicine Gateway (24.2%).

## Discussion

This study presents a cross-disciplinary investigation of practitioner-reported strategies for recurrent or persistent *Candida*-related vulvovaginal presentations in Australia. These presentations pose therapeutic challenges across clinical settings. The respondent cohort reflected the cross-disciplinary nature of this care, comprising both conventional and complementary medicine practitioners, including general practitioners, pharmacists, naturopaths, herbalists, and clinical nutritionists.

A total of 122 different treatments were identified. This breadth may partly reflect overlapping management approaches directed at reducing *Candida* burden or managing associated vulvovaginal symptoms across recurrent or persistent presentations, rather than recommendations confined to recurrent acute infection alone. Across 223 respondents, therapeutic recommendations showed clear disciplinary orientation. Conventional medicines were reported by 43.9% of respondents, most commonly from conventional backgrounds (68.4%), although nearly one-third were contributed by complementary medicine practitioners (31.6%). The conventional agents most frequently cited were azole antifungals. Azoles inhibit 14-α-lanosterol demethylase, disrupting ergosterol synthesis and compromising fungal cell membrane integrity. Nystatin, a polyene reported less frequently in this cohort, binds directly to ergosterol and increases membrane permeability [49]. These established mechanisms underpin the clinical role of these agents in recurrent or persistent *Candida*-related vulvovaginal presentations, as reflected in the treatment selections reported in this study. In contrast, herbal and nutrient-based interventions were predominantly recommended by complementary medicine practitioners (herbal 97.2%; nutrients 78.6%).

Recommendations were not confined strictly within disciplinary boundaries. Among complementary medicine practitioners, boric acid capsules were the most frequently recommended conventional treatment (48.4%). Boric acid is cited in national guidance for azole-resistant or non-albicans *Candida* infections [25, 28]. Experimental data indicate that boric acid inhibits hyphal morphogenesis and disrupts mature biofilms, potentially through cytoskeletal interference and extracellular matrix destabilisation [50], positioning it within recognised management pathways for persistent or recurrent presentations [28, 51].

Probiotics, administered either orally (94.8%) or intravaginally (71.9%), were cited across professional groups. Probiotics have been investigated as both standalone agents and adjuncts to pharmaceutical therapy in vaginal health [52–56]. Clinical trials evaluating oral and intravaginal *Lactobacillus* combinations, administered alone or alongside azole maintenance regimens, have reported rapid vaginal lactobacilli colonisation, symptom resolution, and reduced recurrence rates of infection in women [22, 41, 57, 58]. Intravaginal probiotic formulations are listed on the Australian Register of Therapeutic Goods (ARTG) [59], confirming their status as regulated therapeutic products in Australia. The frequent recommendation of probiotics in this cohort is consistent with research evaluating microbiota-directed strategies in *Candida*-related presentations.

Herbal medicines constituted a substantial proportion of reported treatments, with 69 distinct agents identified, indicating considerable heterogeneity within herbal recommendations. The following examples contextualise frequently reported botanical recommendations, while the wider herbal dataset identifies priorities for future appraisal of clinical evidence, including evidence gaps, effectiveness, and safety data. Frequently cited herbs included *Allium sativum* (garlic), *Handroanthus impetiginosus* (pau d’arco), *Pseudowintera colorata* (horopito), and *Vaccinium macrocarpon* (cranberry), plants with anti-*Candida* activity described primarily in preclinical literature [30, 36, 38, 60–62]. Polygodial in *Pseudowintera colorata (*horopito*)* has demonstrated inhibition of *Candida albicans *in vitro [60], while clinical investigations in women have reported improvements in symptom burden and relapse frequency following use of horopito-containing formulations [31, 63]. β-lapachone from *Handroanthus impetiginosus* (pau d’arco) has similarly shown antifungal activity against azole-resistant *Candida albicans* in experimental models [38].

Beyond direct antifungal effects, several of the herbal medicines identified have also shown immunomodulatory, antioxidant, and cytoprotective properties in experimental and in vivo models [64–71]. The prominence of herbal recommendations suggests that plant-derived therapies remain incorporated within reported RVVC care in this cohort. However, much of the evidence remains preclinical, and rigorous clinical trials are required to determine efficacy, dosing, and appropriate integration of plant-based interventions in RVVC management, alongside pharmacovigilance-oriented appraisal of safety, interactions, suitability, and treatment context.

Nutrient-based and non-prescription product recommendations were reported, including vitamins D and C, N-acetylcysteine (NAC), and oral yoghurt. These agents have been examined in relation to immune and inflammatory mechanisms, host–pathogen interactions, biofilm disruption, and microbiome dynamics in experimental and clinical contexts [42–44, 72–74]. Intravaginal and topically applied interventions included probiotics, yoghurt, vinegar, and commercially available vaginal preparations. Collectively, these approaches form part of the therapeutic repertoire described in this cohort, reflecting the use of systemic and locally applied strategies in reported practice.

Dietary and lifestyle measures featured prominently, with several dietary modifications identified. Among these, reducing sugar and refined carbohydrate intake was frequently reported and aligns with observational studies examining dietary patterns, glucose tolerance and vulvovaginal candidiasis [47, 48, 75–77]. These associations are largely derived from observational data and remain heterogeneous; however, in the present study dietary modification formed part of practitioner-reported management strategies.

Hygiene practices, such as the avoidance of synthetic underwear, front-to-back wiping after urination, and limiting excessive vulval washing, were commonly selected. Observational studies have identified intravaginal douching, tight clothing, and certain sexual behaviours as risk factors for vulvovaginal candidiasis, whereas other practices such as underwear fabric and toileting hygiene have shown inconsistent associations [46, 48]. Although behavioural risk factors have been described, episodes can still occur in the absence of identifiable external triggers, reflecting the multifactorial nature of RVVC and the importance of accommodating individual variability in clinical management.

This study also examined the information sources consulted by healthcare professionals. Conventional practitioners predominantly cited national clinical guidelines including the Therapeutic Guidelines (eTG) and the Australian STI Management Guidelines (ASHM), which synthesise evidence from peer-reviewed trials and expert consensus. Complementary medicine practitioners more commonly consulted peer-reviewed literature, clinical reference databases, and professional association guidance. Both groups drew on multiple forms of evidence, albeit with differing emphasis. These differences may influence how evidence is interpreted and applied across professional contexts, particularly where centralised, condition-specific guidance is limited and reliance on dispersed literature becomes necessary. The variation in information sources consulted may, in part, contribute to the range of treatment recommendations identified in this study.

This study has several limitations. Participants comprised a self-selecting sample, consistent with surveys employing convenience and snowball sampling, where exposure cannot be fully quantified. The predominance of complementary medicine respondents likely reflects the recruitment reach of multi-platform dissemination rather than targeted recruitment. While not generalisable to the wider healthcare workforce, the sample offers insight into clinician-reported treatment strategies. The study was designed to identify the treatments recommended by practitioners in clinical practice, rather than the reasons underpinning each recommendation. This design supported broad capture of treatment patterns across multiple categories while maintaining a feasible questionnaire length and limiting responder burden. However, further treatment-level appraisal of evidence quality, effectiveness, safety, interactions, and pharmacovigilance considerations across reported therapies requires a separate evidence synthesis. Interpretation should also consider that practitioner recommendations may have been informed by differing clinical understandings of recurrent, persistent, or chronic *Candida*-related vulvovaginal presentations. Further work is warranted to examine treatment selection, evidence quality, and application in different clinical contexts. The predominance of female participants suggests several overlapping factors. The Australian complementary medicine workforce is primarily female [78, 79]; women are more likely to participate in health research [80, 81]; and women tend to seek care from female practitioners for intimate conditions such as RVVC [82].

## Conclusion

This study offers a contemporary overview of practitioner-reported RVVC management across healthcare disciplines. The findings demonstrate substantial variation in treatments recommended in practice, including the use of complementary medicine therapies both as primary treatments and alongside conventional care. Further evaluation of these treatments is needed to inform evidence-based guidance that supports practitioner-led decision-making. Well-designed clinical trials of selected complementary medicine interventions are a clear priority to strengthen the evidence base in this area. Greater cross-disciplinary understanding of available treatment approaches and their evidence base may also support safer and more coordinated care for women with recurrent or persistent *Candida*-related vulvovaginal symptoms.

## Supplementary Information

Below is the link to the electronic supplementary material.

Supplementary file1 (PDF 164 KB)

## Data Availability

The datasets generated and/or analysed during the current study are available from the corresponding author on reasonable request.
