# Supplementary material for: Treatment recommendations for recurrent vulvovaginal candidiasis across complementary and conventional disciplines: a national survey of Australian healthcare professionals
Source: Int J Clin Pharm. 2026 May 26;48(5):1926–36. doi: 10.1007/s11096-026-02165-5 (PMC13569548; doi:10.1007/s11096-026-02165-5)
Supplement: Supplementary file 1 [file 11096_2026_2165_MOESM1_ESM.pdf]

Online Resource 1. Supplementary information.

Treatment recommendations for recurrent vulvovaginal candidiasis across complementary and conventional disciplines: a national survey of Australian healthcare professionals

International Journal of Clinical Pharmacy

Rommy Everingham-Gordon, Elizabeth Steels, Cathy J Watson, Kathryn J Steadman.

Corresponding author:
Rommy Everingham-Gordon
School of Pharmacy and Pharmaceutical Sciences
Faculty of Health, Medicine and Behavioural Sciences
The University of Queensland, Brisbane, Queensland, Australia
Email: r.everinghamgordon@uq.edu.au

Additional tables are provided as online resources due to journal limits on the number of tables and figures permitted within the main manuscript. These materials contain full treatment listings and extended results for completeness of reporting.

Supplementary Table S1. Herbal medicines cited by health practitioners as having been recommended for the treatment of RVVC

Predefined survey options	Additional entries identified from practitioner responses (as single agents or in combination)			
Cranberry<br>(*Vaccinium macrocarpon*)	Andrographis<br>(*Andrographis paniculata*)	Coptis<br>(*Coptis chinensis*)	Kochiae Fructus<br>(*Kochia scoparia; Bassia scoparia*)	Pomegranate<br>(*Punica granatum)*
Horopito<br>(*Pseudowintera colorata*)	Anemarrhenae rhizome<br>(*Anemarrhena asphodeloides*)	Da Cheng-Qi-Tang<br>(TCM formulation)	Lavender<br>(*Lavandula angustifolia*)	Poria cocos<br>(*Wolfiporia extensa)*
Rosemary<br>(*Rosmarinus officinalis* –<br>taxonomically updated to<br>*Salvia Rosmarinus)*	Anise oil<br>(*Pimpinella anisum)*	Dang Gui Nan Tong Tang<br>(TCM formulation)	Licorice<br>(*Glycyrrhiza glabra*)	Pulsatilla<br>(*Pulsatilla chinensis*)
Peppermint<br>(*Mentha piperita*)	Agrimony<br>*(Agrimonia eupatoria)*	Dictamni radicis cortex / Chinese dittany root bark<br>(*Dictamnus dasycarpus*)	Long Dan Xie Gan Wan<br>(TCM formulation)	Rehmannia<br>(*Rehmannia glutinosa*)
Curcumin<br>(*Curcuma longa; turmeric*)	Baical skullcap<br>(*Scutellaria baicalensis*)	Echinacea<br>(*Echinacea purpurea*)	Marshmallow<br>(*Althaea officinalis*)	Sage<br>(*Salvia officinalis*)
Chamomile<br>(*Matricaria chamomilla*)	Barberry<br>(*Berberis vulgaris*)	Fennel<br>(*Foeniculum vulgare*)	Mediherb Bacto-Cand GI<br>(formulated product)	Sea Buckthorn<br>(*Hippophae rhamnoides)*
Garlic<br>(*Allium sativum*)	Burr marigold<br>(*Bidens tripartita)*	Fructus Cnidii<br>(*Cnidium monnieri*)	Myrrh<br>(*Commiphora myrrha*)	Shatavari<br>(*Asparagus racemosus*)
Ginger<br>(*Zingiber officinale*)	Calendula<br>(*Calendula officinalis*)	Gentian<br>(*Gentiana lutea*)	Nigella<br>(*Nigella sativa*)	TCM herbs (undefined)
Pau d'arco<br>(*Handroanthus impetiginosus*)	Cangzhu<br>(*Atractylodes lancea;<br>Atractylodes chinensis)*	Green tea<br>(*Camellia sinensis*)	Oregano<br>(*Origanum vulgare*)	Tea tree<br>(*Melaleuca alternifolia*)
St Mary's Thistle<br>(*Silybum marianum*)	Caraway oil<br>(*Carum carvi*)	Golden rod<br>(*Solidago virgaurea*)	Oregon grape<br>(*Berberis aquifolium*)	Triphala<br>(Ayurvedic formulation)
	Cat's claw<br>(*Uncaria tomentosa; Uncaria guianensis*)	Goldenseal<br>(*Hydrastis canadensis*)	Olive leaf<br>(*Olea europaea*)	Thuja<br>(*Thuja occidentalis*)
	Chinese Yam<br>(*Dioscorea opposita*)	Gotu kola<br>(*Centella asiatica*)	Oyster shell<br>(*Ostreae concha*)	Thyme<br>(*Thymus vulgaris*)
	Citrus seed extract<br>(*Citrus* spp.)	Gymnema<br>(*Gymnema sylvestre*)	Phellodendron<br>(*Phellodendron amurense*)	Wormwood<br>(*Artemisia absinthium*)
	Clove<br>(*Syzygium aromaticum)*	Holy Basil<br>(*Ocimum tenuiflorum*)	Pelargonium<br>(*Pelargonium sidoides)*	Yomogi / Japanese mugwort<br>(*Artemisia princeps*)
	Codonopsis<br>(*Codonopsis pilosula*)	Houttuynia<br>(*Houttuynia cordata*)	*Plantago* spp.<br>(species not specified)	

Supplementary Table S2. Nutritional medicines cited by health practitioners as having been recommended for the treatment of RVVC

Predefined survey options	Additional entries identified from practitioner responses (as single agents or in combination)	
Probiotics (oral intake)	Lactulose (oral)	Collagen
Zinc	Prebiotics	D-Mannose
Vitamin D	Iron (supplemental form)	Enzymes (unspecified)
Vitamin C	Vitamin A (supplemental form)	Kefir
Yoghurt (oral intake)	Barley	Lactoferrin
N-acetylcysteine (NAC)	Biotin (supplemental form)	Molybdenum (supplemental form)
	Calcium D-glucarate	Sulforaphane (from cruciferous vegetables)
		Usnea

Supplementary Table S3. Over-the-counter products and home remedies cited by health practitioners as having been recommended for the treatment of RVVC

Predefined survey options	Additional entries identified from practitioner responses (as single agents or in combination)
Moisture replacement (intravaginal application e.g. Aci-Jel Restore, Replens, Vagisil Pro-Hydrate)	Ascorbic acid intravaginal (encapsulated powder)
pH balance wash or gel (intravaginal application e.g. Aci-Jel Balance)	General moisturisers (e.g., Dermeze Treatment ointment, QV Intensive)
Vaginal douche (intravaginal application e.g. Summer’s Eve)	Herbal antifungal topical/external (e.g. Kolorex vaginal care cream and wash)
Intimate wash (external application e.g. Femfresh, Canestan, Vagisil, Summer’s Eve)	Lactic acid intravaginal (e.g., Dr. Wolff's Vagisan Lactic Acid pessaries)
Probiotics (intravaginal application)	Lactulose external/vulval
Yoghurt (intravaginal application)	Lactulose intravaginal
Yoghurt external/vulval	Magnesium external/vulval (e.g., Magnesium bath)
Vinegar intravaginal (e.g. Apple cider vinegar)	MCT oils external/vulval (e.g. coconut oil)
Vinegar external/vulval (e.g. Apple cider vinegar)	MCT oils intravaginal (e.g. coconut oil pessary)
	Vitamin D intravaginal
	Propolis external/vulval
	Colloidal silver
	Hydrogen peroxide

Supplementary Table S4. Dietary exclusions recommended by study participants for the management of RVVC

Dietary exclusions	Respondents recommending diet exclusions n = 178		Conventional medicine practitioners n = 26		Complementary medicine practitioners n = 152	
	n	%	n	%	n	%
Reduce sugary products and refined products (e.g. bread, sweets, pastries, etc)	177	99.4	26	100	151	99.3
Reduction or elimination of alcohol intake	143	80.3	7	26.9	136	89.5
Reduction or elimination of potential food allergens (e.g. dairy, wheat)	115	64.6	5	19.2	110	72.4
Remove yeast/yeast foods & products	9	5.1	0	0.0	9	5.9
Remove fungi (e.g. mushrooms, truffles)	5	2.8	0	0.0	5	3.3
Remove specific irritants (e.g. hot spicy foods, allium plants, condiments, coffee)	5	2.8	0	0.0	5	3.3
Remove fermented foods and products	4	2.2	1	3.8	3	2.0

Supplementary Table S5. Lifestyle and hygiene-related practices recommended by study participants for the management of RVVC

Lifestyle and hygiene practices	Respondents recommending lifestyle changes n = 207		Conventional medicine practitioners n = 56		Complementary medicine practitioners n = 151	
	n	%	n	%	n	%
Avoid wearing synthetic underwear/undergarments	182	87.9	46	82.1	136	90.1
Avoid excessive washing of vulval area (not intercourse related)	144	69.6	37	66.1	107	70.9
Wipe vaginal area backwards after urination	133	64.3	28	50.0	105	69.5
Avoid intravaginal douching	103	49.8	35	62.5	68	45.0
Avoidance of sexual intercourse during treatment	96	46.4	14	25.0	82	54.3
Treatment of sexual partner (includes education, testing and treatment)	16	7.7	2	3.6	14	9.3
Avoid local irritants (e.g. latex condoms, antiseptics, daily liners, synthetic vaginal products, synthetic lubricants, baby wipes, shower gels, bubble baths, soaps, fabric softeners, harsh laundry detergents)	15	7.2	8	14.3	7	4.6

Supplementary Table S6. Information resources consulted by study participants providing treatment recommendations for RVVC

Resource	Respondents selecting an information resource n = 208		Conventional medicine practitioners n = 59		Complementary medicine practitioners n = 149	
	n	%	n	%	n	%
Journal articles	135	65.0	25	42.4	110	73.8
Textbooks	98	47.1	9	15.3	89	59.7
Colleagues	89	42.8	13	22.0	76	51.0
Undergraduate/Post graduate studies	79	38.0	9	15.3	70	47.0
Information from product manufacturers	76	36.5	4	6.8	72	48.3
Natural Medicines Database	70	33.7	1	1.7	69	46.3
Therapeutic Guidelines (eTG)	53	25.5	34	57.6	19	12.8
Professional Association or Society	53	25.5	12	20.3	41	27.5
Integrative Medicine Gateway Database	37	17.8	1	1.7	36	24.2
NPS MedicineWise (National Prescribing Service) www.nps.org.au	30	14.4	13	22.0	17	11.4
Australian STI Management Guidelines for Use in Primary Care (ASHM)	27	13.0	19	32.2	8	5.4
